# Malignant osteopetrosis of infancy: A case report

**DOI:** 10.1016/j.ijscr.2024.110206

**Published:** 2024-08-22

**Authors:** Fatemeh Avijgan, Farzaneh Abbasian, Mahdie Dehnavi, Zohreh Sarchahi

**Affiliations:** Department of Nursing and Midwifery, Neyshabur University of Medical Sciences, Neyshabur, Iran

**Keywords:** Infant, Osteoporosis, Anaemia, Case report

## Abstract

**Introduction:**

Osteopetrosis is a genetic bone disease whose main feature is the function of osteoclasts. This rare disorder affects one in every 250,000 live births. In terms of pathophysiology, osteopetrosis is divided into four types from mild to severe forms of the disease. We report of a case report of malignant osteopetrosis of infancy.

**Case presentation:**

A 3-month-old infant weighing 5 kg was hospitalized with complaints of fever, anaemia, and thrombocytopenia. He was the fourth child of the family, three other children of the family have died due to osteoporosis. According to the history of the disease in the family and the symptoms, tests and radiographic results, the diagnosis of osteopetrosis has been made for the child.

**Discussion:**

It involves an autosomal recessive mode of inheritance and manifests as diffuse osteosclerosis. Benign autosomal dominant osteopetrosis is a common form of the disease that is without symptoms and is diagnosed by random radiography. About half of the cases are characterised by fracture or osteomyelitis of the lower jaw Congenital osteopetrosis is a severe and malignant form of the disease that occurs in infancy.

**Conclusion:**

Considering the high mortality in osteopetrosis patients and the psychological burden and significant economic concern that comes with it. There is a need for timely diagnosis and treatment as soon as possible.

## Highlights

• Osteopetrosis is a genetic bone disease whose main feature is the function of osteoclasts.
• The patient was the fourth child of the family with osteoporosis based on the history of the disease in the family and the symptoms, tests and radiographic results.
• A bone marrow transplant was performed for the patient and it was unsuccessful.

## 1 Introduction

Osteopetrosis is a genetic bone disease whose main feature is a defect in the function of osteoclasts [1,2], which is characterised by an increase in bone density and bone deformity in the clinic [3]. Osteo means bone and petros means stone [4]. Elberschönberg, a radiologist, was the first to describe this disease in 1904 in a 26-year-old man with a clinical picture of severe scoliosis and pathological fractures [4,5]. This disorder is rare and affects 1 in every 250,000 live births [6,7]. According to the severity of the disease, it can be divided into malignant “infantile autosomal recessive” and benign type “adult autosomal dominant” and intermediate type [1,3]. Its malignant type is common in infancy and rapidly deteriorates. River and leads to death in the first few years [8,9].

The definite diagnosis of this disease is severe sclerosis in the pelvic bones and the vertebral column, and the treatment is supportive and symptomatic [4]. Patients with the benign type have a normal lifespan, but there is a possibility of long bone fractures and bone deformities in them [8,10]. The clinical manifestations are related to the increase in bone density due to the decrease in the activity of osteoclasts [11]. Children have certain phenotypic features such as macrocephaly and wide face, delayed teeth growth, multiple bone fractures, short stature and mental retardation [6,12]. Nasal congestion is caused by underdevelopment of the mastoid and nasal sinuses [6]. Neurological clinical manifestations are due to excessive growth of the skull bones and narrowing of the cranial hole, which leads to compression of the optic and auditory nerves and causes blindness, hearing loss, and facial paralysis [13,14].

Children with osteopetrosis are at risk for seizures due to secondary hypocalcemia and hyperparathyroidism [6]. The failure is caused by the reduction of bone marrow space [14] and leads to compensation of extramedullary hematopoiesis in various organs such as the spleen and liver, and ultimately leads to hepatosplenomegaly, anaemia, thrombocytopenia, granulocytopenia and recurrent infections in patients [9,10]. Osteopetrosis imposes a significant psychological and economic burden on families with significant mortality [8]. Diagnosis of this disease is challenging and often delayed or misdiagnosed. Therefore, it is important for doctors to be familiar with this rare disease [11].

## 2 Case report

A 3-month-old patient (H-B) weighing 5 kg was admitted with a complaint of fever and diagnosis of anaemia and thrombocytopenia. According to the medical record, the child is a known case of osteopetrosis. He is the fourth child in the family, and three other children in the family have died due to the same disease. Parents are related. Lung and heart auscultation are normal. In appearance, the forehead is prominent. Eye movements are abnormal. Patient (H-B) was born at full term and had a normal birth. He had a history of hospitalization during infancy, and due to the family history and calcium deficiency in the patient, the diagnosis of osteopetrosis was made for the child. Atrophy of the optic nerve and blindness have been diagnosed for the child since he was 2 months old. The patient underwent primary treatment according to CBC and PLT, PC was transfused and interferon gamma ampoule was injected. The patient was discharged due to the improvement of anaemia symptoms and the order to inject interferon gamma ampoules 3 times a week and calcitriol tablets and repeat the CBC one week later. In addition, the patient was a candidate for a bone marrow transplant and it done for patient.

## 3 Laboratory tests

RBC: 2/85 HB: 8/3 HCT 28/5 PLT: 45000 MCV: 100 MCHC: 29.12 WBC: 19200, SGOT: 98 SGPT:15 ALP:1397, CRP:23.

## 4 Vital signs

T:37 RR:69 HR: 160 SPO2: 97 % with oxygen therapy 5 L/MIN.

Two units of platelets and 80 ml of pixels were transfused for the patient. He underwent antibiotic therapy and the patient was discharged. With the follow-ups, the child had frequent hospitalizations with symptoms of anaemia and infection and was discharged after receiving treatment and partial recovery. At the age of 24 months, the patient was hospitalized again with symptoms of respiratory distress, decreased platelets, leukocytosis, and poor general condition in the intensive care unit. Due to the severity of the symptoms and the progress of the disease, he did not respond to the treatment and died. The work has been reported in line with the SCARE criteria [15].

## 5 Discussions

In this case, the baby presented with symptoms of fever, thrombocytopenia and anaemia. In the study of Khorashadizadeh et al., a case was reported with early symptoms of fever, thrombocytopenia and anaemia along with hepatosplenomegaly at the age of 2 months [4]. These symptoms indicate a lack of bone marrow function. In terms of pathophysiology, four types of osteopetrosis have been described:

A- Severe neonatal-malignant type associated with mutations in TCIRG and C1CN7 genes. It starts in the womb and is diagnosed in the first year of birth. Features are severe and patients usually do not survive more than 20 years.
b. Osteopetrosis with renal tubular acidosis and cerebral calcification associated with carbonic anhydrase II (CA II) deficiency and mutations in the gene encoding CA II protein. It appears in early childhood but may regress spontaneously.
J. A benign autosomal dominant variant whose causative gene remains to be identified on chromosome 11q12–13. Patients usually present with no symptoms and are diagnosed on routine radiographs.
d. Moderate autosomal recessive type with clinical features of neonatal or asymptomatic manifestations. It involves an autosomal recessive mode of inheritance and manifests as diffuse osteosclerosis [[16], [17]]. Benign autosomal dominant osteopetrosis (c) is a common form of the disease that is without symptoms and is diagnosed by random radiography. About half of the cases are characterised by fracture or osteomyelitis of the lower jaw [18] Congenital osteopetrosis is a severe and malignant form of the disease that occurs in infancy. This disease is transmitted in an autosomal recessive form. The symptoms of the disease are blindness, deafness, hydrocephalus, anaemia, hepatosplenomegaly, and bone fractures. In the radiograph of the bones, an increase in generalized thickness is seen. The cranial nerves that pass through the bones, such as the nerves of sight and hearing, are under pressure due to the increase in bone growth, causing blindness and deafness in these patients. Intrauterine diagnosis is possible through radiography. These patients usually die by the age of two due to anaemia, bleeding and infection [18].

We have to attention that the clinical symptoms based on the situation of patient can be different. Another case report showed the most of symptoms were hepatosplenomegaly, axial hypotonia, and visual impairment. Blood investigation revealed pancytopenia and hypocalcemia which were different from present case report symptoms [19].

## 6 Conclusion

According to the history and clinical symptoms, radiography and tests, the case reported in this study is a malignant type of infancy. A bone marrow transplant was performed for the patient and it was unsuccessful. With timely diagnosis and starting the treatment as soon as possible, the complications of the disease can be prevented and bone marrow transplantation can be done as soon as possible. Although there is a possibility of failure in the treatment with bone marrow transplant. Researchers hope that by determining the gene of the disease, it will be possible to treat this disease through gene therapy in the future.

## Consent

Written informed consent was obtained from the patient's parents/legal guardian for publication and any accompanying images. A copy of the written consent is available for review by the Editor-in-Chief of this journal on request.

## Ethical approval

The Study Protocol was approved by the Research and Ethics Committee of Neyshabur University of Medical Sciences (IR.NUMS.REC.1402.010).

## Funding

This research did not receive any specific grant from funding agencies in the public, commercial, or not-for-profit sectors.

## Author contribution

Fatemeh Avijgan and Farzaneh Abbasian has designed the concept of the study, literature review, Data Collection and analysis. Dr. Mahdie Dehnavi and Zohreh Sarchahi has contribution in study concept design, treatment of the patient and manuscript writing.

## Guarantor

Zohreh Sarchahi.

## Research registration number

Not applicable.

## Conflict of interest statement

The authors report no declarations of interest.
